# TRIM29 inhibits PRRSV replication by targeting nsp11 for degradation

**DOI:** 10.1128/jvi.01512-25

**Published:** 2025-11-18

**Authors:** Wei Wen, Zhenghong Xue, Yi Lu, Yuhang Liu, Wenqiang Wang, Zhenbang Zhu, Xiangdong Li

**Affiliations:** 1 Jiangsu Co-innovation Center for Prevention and Control of Important Animal Infectious Diseases and Zoonoses, College of Veterinary Medicine, Yangzhou University 38043, Yangzhou, People’s Republic of China; 2 Joint International Research Laboratory of Agriculture and Agri-Product Safety, Ministry of Education of China, Yangzhou University 38043, Yangzhou, People’s Republic of China; University of Kentucky College of Medicine, Lexington, Kentucky, USA

**Keywords:** PRRSV, nsp11, TRIM29, proteasome degradation, endonuclease activity site

## Abstract

Ubiquitination plays critical roles in viral infections. This study demonstrates that porcine reproductive and respiratory syndrome virus (PRRSV) endoribonuclease nsp11 undergoes K48-linked polyubiquitination specifically at the conserved catalytic residue lysine 173 (K173) during viral infection. This modification targets nsp11 for degradation via the ubiquitin-proteasome system (UPS), as evidenced by the profound stabilization of a ubiquitination-deficient K173R mutant. Remarkably, this ubiquitination mechanism targeting the endonuclease active site is evolutionarily conserved across most arteriviruses, including simian hemorrhagic fever virus and equine arteritis virus, with mouse lactate dehydrogenase-elevating virus being an exception. We further identify the host E3 ubiquitin ligase TRIM29 as a key regulator that binds PRRSV nsp11 via its coiled-coil domain and specifically promotes its K48-linked ubiquitination and subsequent proteasomal degradation. TRIM29-mediated degradation of nsp11 counteracts nsp11’s suppression of interferon (IFN-β) and interferon-stimulated gene production. Consequently, TRIM29 significantly inhibits PRRSV replication. Collectively, these findings uncover a conserved UPS-mediated regulatory mechanism targeting a critical arteriviral endonuclease and demonstrate TRIM29 as a potent host restriction factor that antagonizes PRRSV immune evasion by degrading nsp11.

**IMPORTANCE:**

This study reveals that porcine reproductive and respiratory syndrome virus (PRRSV) nsp11 undergoes K48-linked polyubiquitination at catalytic residue K173, triggering ubiquitin-proteasome system (UPS)-mediated degradation, a mechanism conserved in most arteriviruses. The host E3 ligase TRIM29 binds nsp11 via its coiled-coil domain, catalyzing this ubiquitination to degrade nsp11. This counteracts nsp11’s suppression of interferon (IFN-β)/interferon-stimulated gene production and inhibits PRRSV replication. These findings identify TRIM29 as a key host restriction factor that disrupts viral immune evasion by targeting a conserved arteriviral endonuclease via the UPS.

## INTRODUCTION

The porcine reproductive and respiratory syndrome virus (PRRSV), a member of the Arteriviridae family, represents a significant pathogen that has caused huge economic loss to the global swine industry (1, 2). Despite sharing an ancient evolutionary lineage with coronaviruses, arteriviruses have evolved distinct molecular and biological characteristics that set them apart from other positive-sense RNA viruses (3, 4). The arterivirus family comprises several notable members including equine arteritis virus (EAV), simian hemorrhagic fever virus (SHFV), and mouse lactate dehydrogenase-elevating virus (LDV), along with numerous newly discovered simian variants (5). PRRSV infection manifests as a devastating disease syndrome characterized by reproductive failure in breeding herds and severe respiratory distress in growing pigs, resulting in annual economic losses exceeding $2.5 billion worldwide (6, 7). While vaccination remains the primary control strategy, the remarkable genetic plasticity of PRRSV, particularly among circulating strains in China, has rendered most commercial vaccines ineffective due to rapid viral evolution and immune escape mechanisms (8, 9).

PRRSV possesses a 15.4 kb single-stranded RNA genome containing at least 10 open reading frames (ORFs) (10). The 5′-proximal ORF1a/ORF1b region spans approximately 80% of the viral genome and encodes two large replicase polyproteins (pp1a and pp1ab) that undergo extensive proteolytic processing (11). The pp1ab polyprotein gives rise to four nonstructural proteins (nsp9-12) that form the core viral replication-transcription complex (12). Among these, nsp11 is the only viral factor possessing endoribonuclease activity, a function mediated by its C-terminal EndoU domain. This domain is conserved across nidoviruses and shares distant homology with the *Xenopus laevis* XendoU ribonuclease (13, 14). Structural elucidation reveals that PRRSV nsp11 adopts an unusual asymmetric dimeric configuration, contrasting sharply with the hexameric architecture observed in coronavirus nsp15 homologs (15). The enzyme’s uridylate-specific cleavage activity resides in subdomain A, while subdomain B maintains structural integrity and potentially mediates regulatory interactions (16). PRRSV is an immunosuppressive pathogen that weakens innate immune responses, particularly by suppressing type I interferons (IFNs) (17, 18). A key factor in this immune evasion is NSP11, which possesses nidovirus-like NendoU activity critical for viral replication and host innate immune suppression (19).

Ubiquitination represents a fundamental post-translational modification that orchestrates virtually all aspects of cellular physiology through dynamic protein regulation (20). This versatile modification system achieves its remarkable functional diversity through ubiquitin, a highly conserved 76-amino acid protein containing seven lysine residues (K6, K11, K27, K29, K33, K48, and K63), which forms topologically distinct polymeric chains (21). Among these, K48-linked polyubiquitin chains serve as the canonical signal for proteasomal degradation, while K63-linked chains typically modulate protein-protein interactions and subcellular trafficking (22). The ubiquitination cascade is mediated by a hierarchical enzymatic system comprising E1 activating enzymes, E2 conjugating enzymes, and E3 ligases, with the latter conferring substrate specificity through direct recognition of target proteins (23). Notably, the ubiquitin-proteasome system (UPS) has emerged as a critical battlefield in host-pathogen interactions, with numerous studies demonstrating how viral pathogens exploit or are restricted by host ubiquitination machinery during infection (24).

Functioning as cell-intrinsic antiviral effectors, several TRIM proteins employ diverse strategies to inhibit virus replication. A common mechanism involves their E3 ligase activity, which directs viral proteins for degradation via the ubiquitin-proteasome pathway (25, 26). A key example is TRIM5, which not only targets the tick-borne encephalitis virus protease for ubiquitin-dependent degradation but also restricts primate retroviruses through a proteasome-independent mechanism by binding viral capsids (27). Beyond TRIM5, other family members mediate the degradation of viral proteins from a broad spectrum of viruses, including influenza A virus and enteroviruses (28, 29). TRIM29 belongs to the TRIM family of proteins that function as crucial regulators of diverse cellular processes ranging from cell cycle progression to innate immunity (30, 31). Unlike most TRIM proteins that possess intrinsic E3 ubiquitin ligase activity through their RING domains, TRIM29 has evolved alternative regulatory mechanisms despite lacking this canonical domain (32). Emerging evidence positions TRIM29 as a key modulator of protein turnover, capable of directing the Lys48-linked polyubiquitination and subsequent proteasomal degradation of critical immune regulators, including NEMO and STING (33). This activity effectively suppresses type I interferon responses and inflammatory cytokine production, establishing TRIM29 as an important immune checkpoint molecule. However, its potential role in antiviral defense against RNA viruses, particularly in the context of arterivirus infection, remains completely unexplored.

Our investigation reveals a novel host antiviral mechanism wherein the arteriviral endoribonuclease nsp11 undergoes K48-linked polyubiquitination at a critical catalytic residue, targeting it for proteasomal degradation. Through systematic biochemical and virological analyses, we identify TRIM29 as the E3 ubiquitin ligase responsible for this post-translational modification of PRRSV nsp11. Functional studies demonstrate that TRIM29-mediated degradation of nsp11 substantially attenuates viral replication kinetics. Collectively, our work provides fundamental insights into the intricate host-virus interplay governing PRRSV pathogenesis while revealing potential therapeutic targets for intervening in arterivirus infections.

## RESULTS

### PRRSV nsp11 undergoes ubiquitination during viral infection

**Fig 1:**
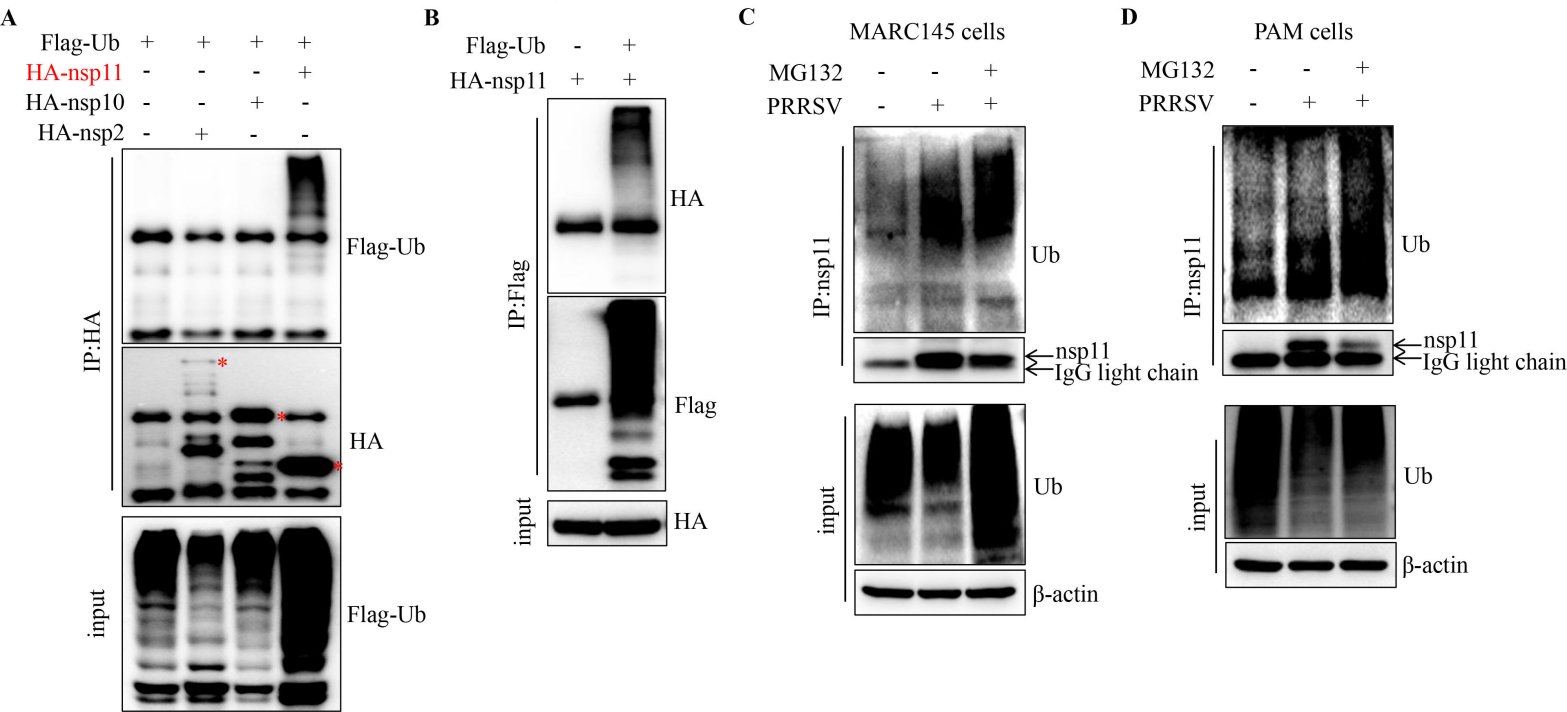
PRRSV nsp11 undergoes ubiquitination during viral infection. (**A**) HEK293T cells were transfected with vector, HA-nsp2, HA-nsp10, or HA-nsp11 along with Flag-Ubi for 30 h, and then the cells were collected for co-immunoprecipitation (Co-IP) analysis. (**B**) HEK293T cells were transfected with vector or Flag-Ubi along with HA-nsp11 for 30 h, and then the cells were collected for Co-IP analysis. (**C**) MARC145 cells were infected with 0.1 MOI PRRSV for 36 h and then treated with MG132 for 12 h. The cells were collected for Co-IP analysis. (**D**) PAM cells were infected with 0.1 MOI PRRSV for 12 h and then treated with MG132 for 12 h. The cells were collected for Co-IP analysis.

Ubiquitination plays a pivotal role in viral infections, with many viruses exploiting this post-translational modification to enhance replication or evade host immune responses (34, 35). While ubiquitination of viral enzymes and cofactors can promote genome replication, host cells also utilize ubiquitination to degrade viral proteins or amplify innate immune defenses. During PRRSV infection, viral RNA synthesis relies on the precise assembly of replication and transcription complexes (RTCs). To determine whether PRRSV RTC components undergo ubiquitination, we co-expressed core RTC proteins with ubiquitin. Notably, we observed robust ubiquitination of nsp11 (Fig. 1A and B). Consistent with a previous study, we also found that PRRSV nsp12 underwent ubiquitination (data not shown). To validate these findings in the context of infection, we performed ubiquitination assays in PRRSV-infected MARC-145 and porcine alveolar macrophage (PAM) cells, with or without the proteasome inhibitor MG132. nsp11, migrating at high molecular weight, was covalently linked to polyubiquitin chains in PRRSV-infected cells, which was further enhanced upon MG132 treatment (Fig. 1C and D), suggesting that ubiquitination occurs during viral replication. Collectively, our results demonstrate that PRRSV nsp11 undergoes ubiquitination, highlighting its potential role in viral replication or host-pathogen interactions.

### PRRSV nsp11 is modified by K48-linked ubiquitin chain at K173

**Fig 2:**
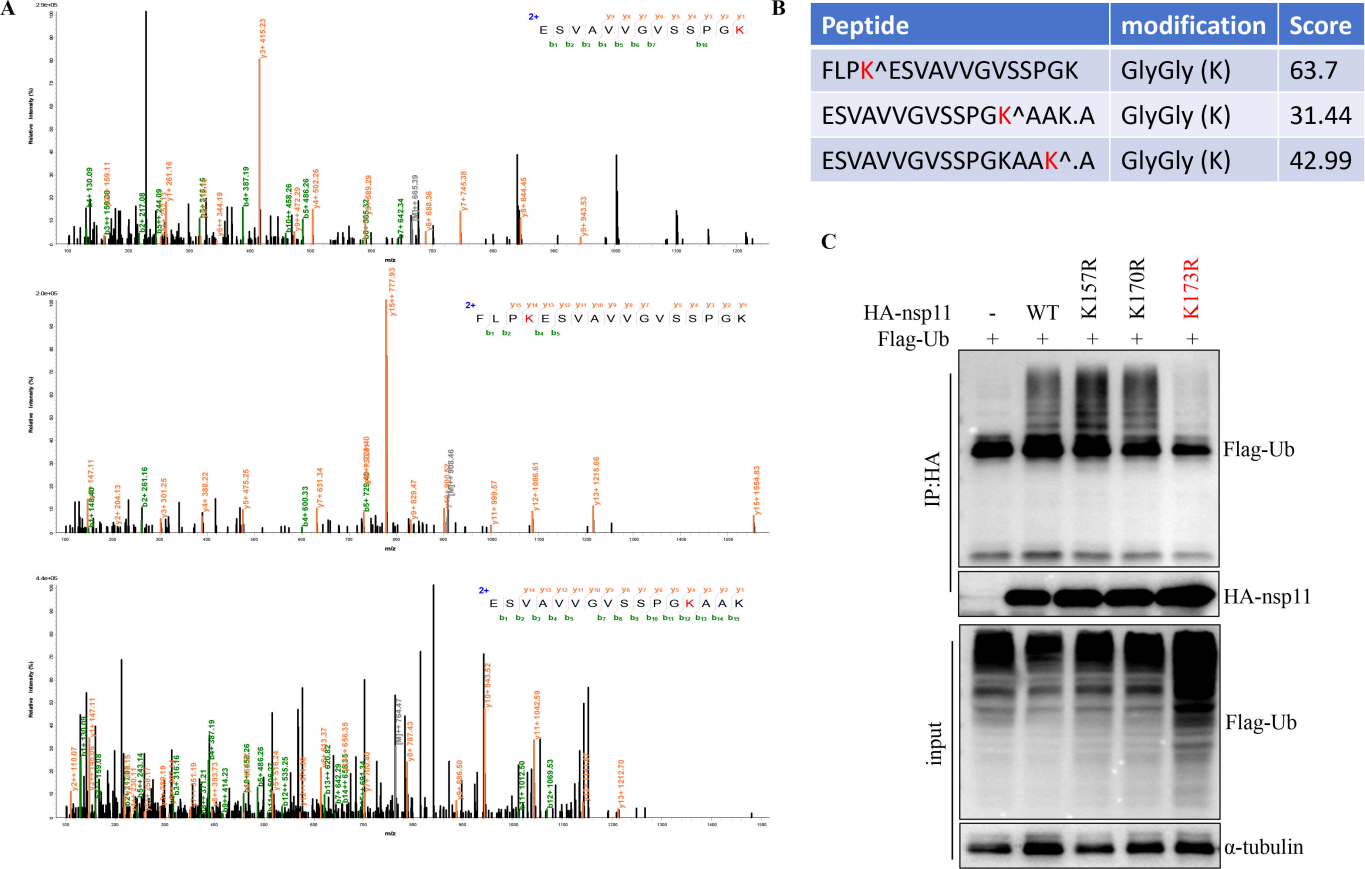
PRRSV nsp11 is ubiquitinated at K173. (**A and B**) Ubiquitinated peptides identified by mass spectrometry. HEK293T cells were transfected with plasmids encoding nsp11. After 30 h, cells were harvested and lysed, and whole-cell extracts were used for immunoprecipitation. Eluted fractions were digested with trypsin and analyzed by MS. (**C**) HEK293T cells were transfected with vector, HA-nsp11, or its mutants along with Flag-Ubi for 30 h, and then the cells were collected for Co-IP analysis.

To map the ubiquitination sites on PRRSV nsp11, we enriched ubiquitinated proteins by immunoprecipitating ectopically expressed nsp11 in the presence of MG132 and subjected the samples to trypsin digestion followed by mass spectrometry (MS). This approach revealed three nsp11-derived peptides (K157, K170, and K173) carrying diglycine modifications, a hallmark of ubiquitinated lysine residues post-trypsin digestion (Fig. 2A and B). To further verify these ubiquitination sites, we constructed different mutants of nsp11 bearing single Lys (K)-to-Arg (R) substitutions and performed immunoprecipitation assays. As expected, multiple ubiquitin chains were detected in the nsp11 immunoprecipitates (Fig. 2C). Remarkably, among the three single K-to-R mutants, only the K173R mutant completely lost ubiquitination signals (Fig. 2C), demonstrating that lysine 173 is the indispensable site for nsp11 ubiquitination. The K173 site is one of the endoribonuclease active sites in PRRSV nsp11 and is essential for viral replication (16).

**Fig 3:**
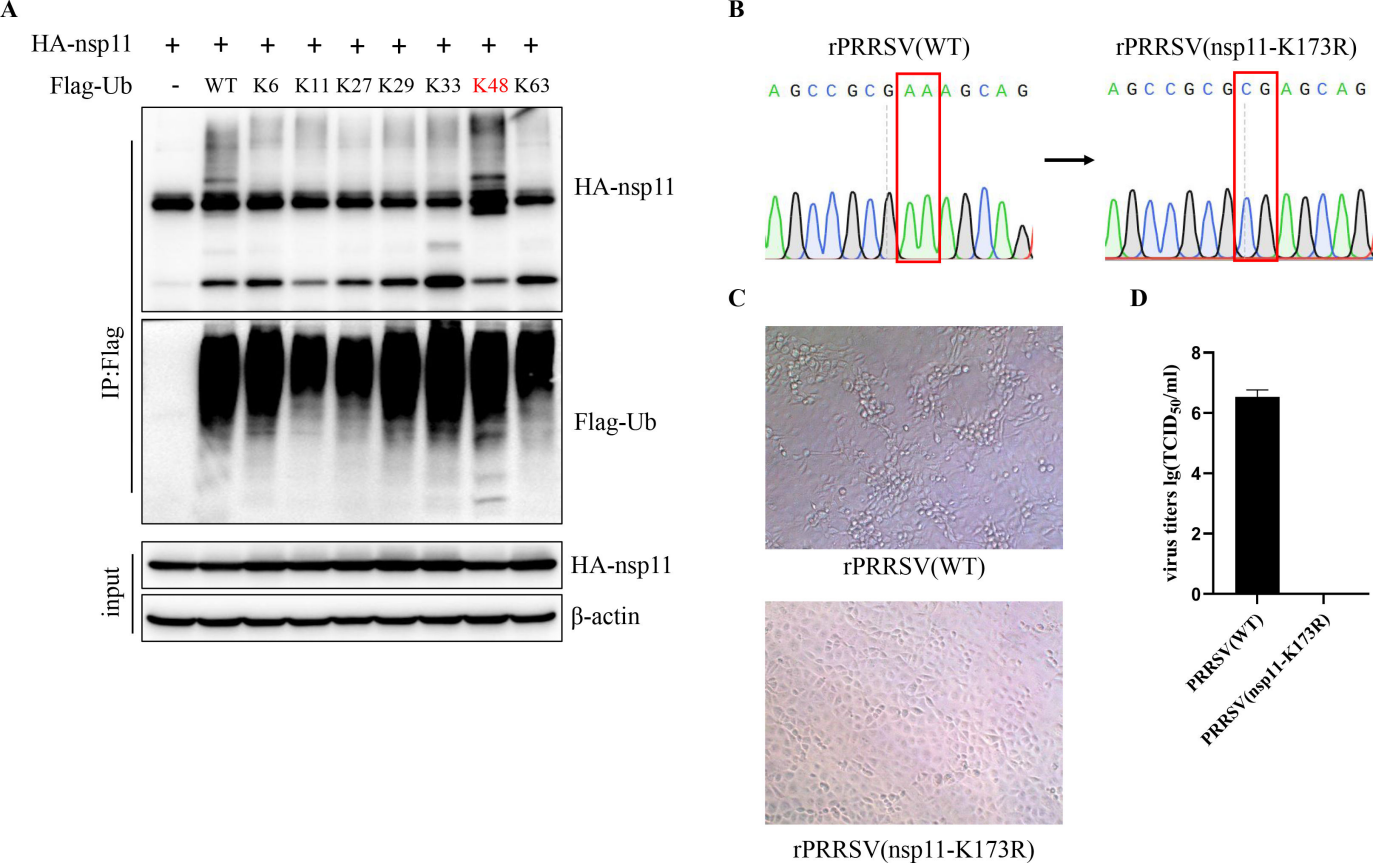
PRRSV nsp11 undergoes K48-linked ubiquitination. (**A**) HEK293T cells were transfected with nsp11 and either wild-type Ub (Ub-WT) or Ub mutants for 30 h before harvesting. Lysates were subjected to immunoprecipitation, followed by western blot analysis of both total lysates and immunoprecipitated samples. (**B**) DNA sequencing of wild-type and mutant nsp11 in PRRSV cDNA clones. (**C and D**) Rescue of recombinant viruses. Briefly, HEK293T cells seeded in 12-well plates were transfected with 2 µg plasmid using Lipofectamine 3000, following the manufacturer’s instructions. At 48 h post-transfection, the culture supernatant was collected as passage zero (P0) virus. The P0 virus was then passaged twice in MARC-145 cells. Subsequently, MARC145 cells were infected with the passage three (P3) virus for 48 h, and cytopathic effects were visualized by electron microscopy (**C**). P3 virus titer was measured by TCID_50_ assay (**D**).

Polyubiquitination can be mediated through any of ubiquitin’s seven lysine residues, generating structurally distinct ubiquitin chains that determine the functional outcome for modified proteins. To define the chain linkage specificity of nsp11 ubiquitination, we performed *in vitro* ubiquitination assays using a panel of ubiquitin mutants where all but one lysine residue were mutated (K6, K11, K27, K29, K33, K48, or K63). Notably, nsp11 ubiquitination was exclusively observed when using the K48-linked ubiquitin mutant (Fig. 3A). To assess the biological significance of nsp11 ubiquitination during PRRSV infection, we engineered a recombinant PRRSV containing the nsp11-K173R mutation via reverse genetics (Fig. 3B). While wild-type virus produced characteristic cytopathic effects (CPEs) upon serial passage, the K173R mutant virus failed to induce detectable CPEs after three passages (Fig. 3C) and no K173R mutant virus can be detected using TCID_50_ assay (Fig. 3D), demonstrating that the K173R mutation is lethal for viral recovery owing to nsp11’s deficient endoribonuclease activity, which is consistent with previous study (36).

### Most arterivirus nsp11 can be ubiquitinated at its endonuclease activity site

**Fig 4:**
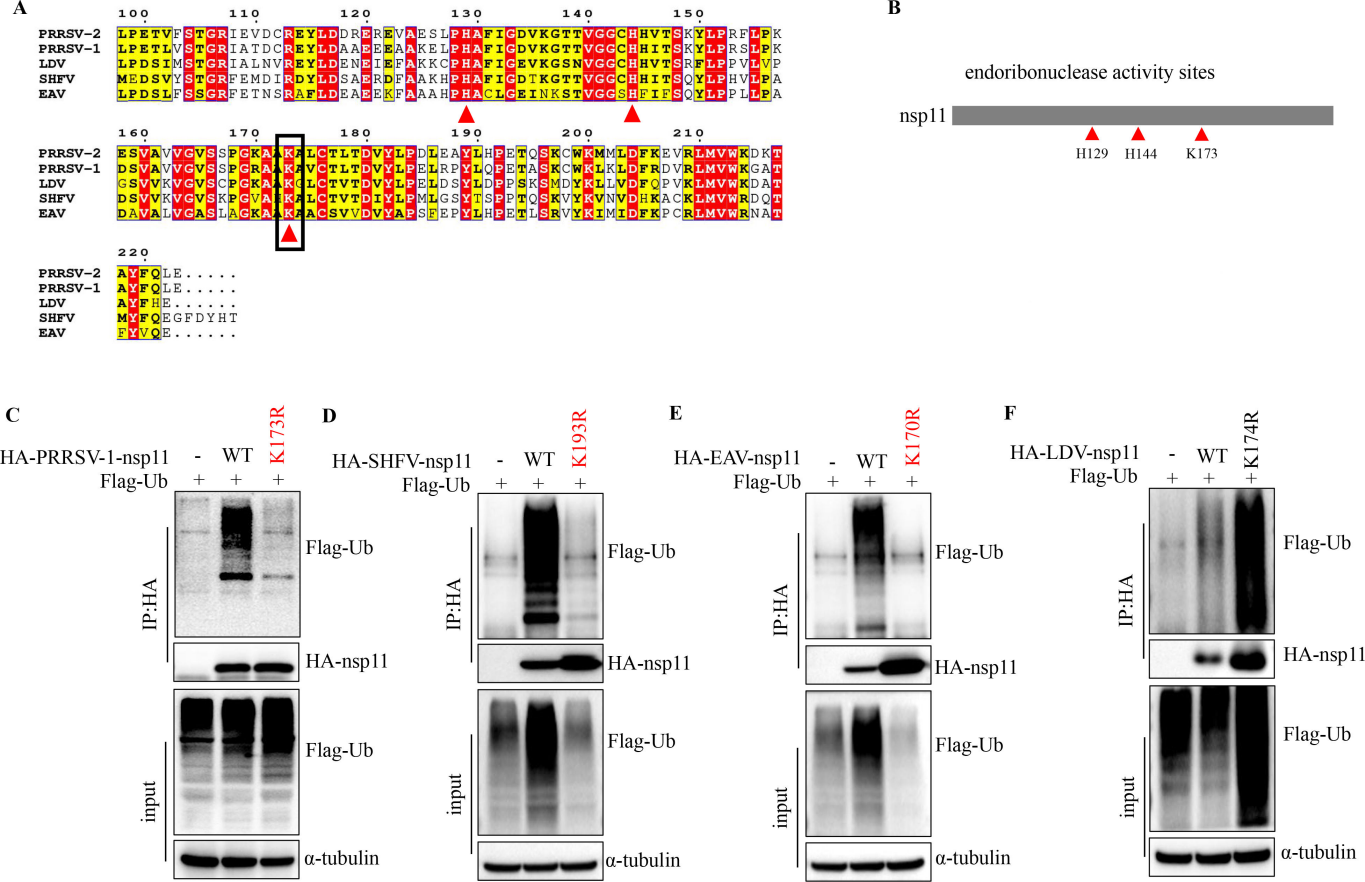
Arterivirus nsp11 is ubiquitinated at its endonuclease activity site. (**A and B**) Sequence alignment of arterivirus nsp11. The red triangles indicate the endoribonuclease active sites of arterivirus nsp11, while the black boxes mark its ubiquitination sites. (**C–F**) HEK293T cells were transfected with ubiquitin along with vector, arterivirus nsp11, or their mutant for 30 h before harvesting. Lysates were subjected to immunoprecipitation, followed by western blot analysis of both total lysates and immunoprecipitated samples.

Despite low overall sequence conservation among arteriviral nsp11 proteins, the K173 residue is strictly conserved and forms an essential part of the endonuclease active site (Fig. 4A and B). Based on this structural constraint, we examined the conservation of nsp11 ubiquitination across arteriviruses and its potential specificity for the K173 residue. Intriguingly, ubiquitination was detected in all tested nsp11 homologs (Fig. 4C through F). In PRRSV-1, SHFV, and EAV, mutagenesis analyses confirmed that ubiquitination occurs at the catalytic residue within the endonuclease active site, precisely recapitulating the K173 modification observed in arteriviruses. Notably, LDV nsp11 constituted an exception, with ubiquitination mapped to a distinct residue (Fig. 4C through F), suggesting potential lineage-specific divergence in regulatory mechanisms. Together, these findings uncover a remarkable evolutionary conservation of nsp11 ubiquitination among most arteriviruses, with modification predominantly targeting catalytic residues critical for endonuclease function.

### Arterivirus nsp11 undergoes ubiquitin-proteasome-mediated degradation

**Fig 5:**
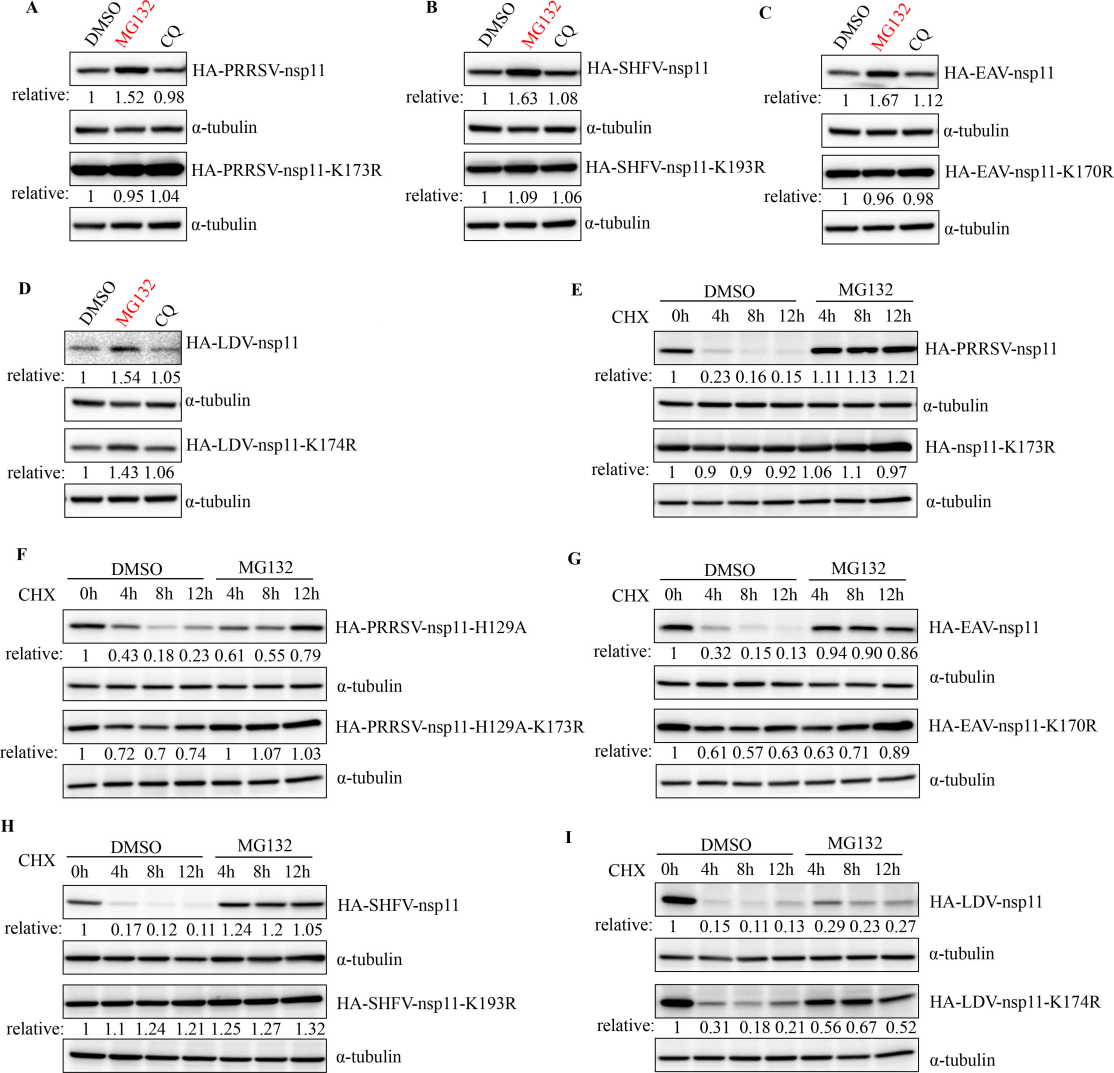
Arterivirus nsp11 undergoes ubiquitin-proteasome-mediated degradation. (**A–D**) HEK293T cells were transfected with arterivirus nsp11 or its mutant for 24 h, and then treated with 10 µM MG132 or 50 mM CQ for 10 h. The cells were collected for western blot. (**E–I**) HEK293T cells were transfected with arterivirus nsp11 or its mutant for 24 h, and simultaneously treated with 50 µg/mL of CHX and 10 µM MG132 prior to being harvested at the indicated time points. The cells were collected for western blot.

Given that PRRSV nsp11 is predominantly modified by K48-linked polyubiquitin chains that are typically associated with protein stability, we sought to systematically investigate whether the turnover of this viral protein is mechanistically regulated through ubiquitin-dependent mechanisms. To definitively map the degradation pathway, we employed pharmacological dissection using chloroquine (CQ) to inhibit autophagic flux and MG132 to block proteasomal activity. As shown in Fig. 5A through D, MG132 treatment caused dramatic accumulation of all arterivirus nsp11, whereas CQ had no measurable effect on protein levels. The ubiquitination-site mutants (with the noted exception of LDV nsp11) showed minimal response to either inhibitor. These results indicated that nsp11 was degraded by the ubiquitin-proteasome system. We also employed a cycloheximide (CHX)-based half-life assay to investigate the involvement of the ubiquitin-proteasome system in the degradation of nsp11. Quantitative immunoblotting revealed striking differential stability profiles: while wild-type nsp11 exhibited progressive degradation with a half-life consistent with active proteasomal targeting, the K173R mutant demonstrated remarkable resistance to decay, maintaining near-complete stability throughout the experimental timeframe (Fig. 5E). Notably, because residue K173 is involved in both ubiquitination and the protein’s endonucleolytic activity, which may confound stability assays, we designed complementary experiments using two additional mutants: an endonuclease-dead variant (nsp11-H129A) and a double mutant with both enzymatic inactivation and ubiquitination deficiency (nsp11-H129A-K173R). This orthogonal approach yielded results fully congruent with our initial observations: the H129A mutant displayed degradation kinetics indistinguishable from wild-type nsp11, whereas the H129A-K173R double mutant recapitulated the stabilization phenotype observed with the single K173R mutation (Fig. 5F), thereby conclusively excluding the effect of nsp11 endonuclease activity on its stability. To establish whether this regulatory paradigm extends across the arterivirus genus, we performed comparative stability analyses on nsp11 homologs from related viruses. Intriguingly, whereas all tested arterivirus nsp11 proteins were degraded in a proteasome-sensitive manner and their ubiquitination-site mutants were stabilized, LDV nsp11 proved an exception: its degradation pattern suggested reliance on a different ubiquitination site (Fig. 5G through I). Collectively, these multifaceted experiments establish that arterivirus nsp11 is selectively targeted for destruction via the ubiquitin-proteasome system, while LDV appears to have evolved distinct regulatory mechanisms.

### TRIM29 induces proteasomal degradation of PPRRSV nsp11 by promoting its K48-linked ubiquitination

**Fig 6:**
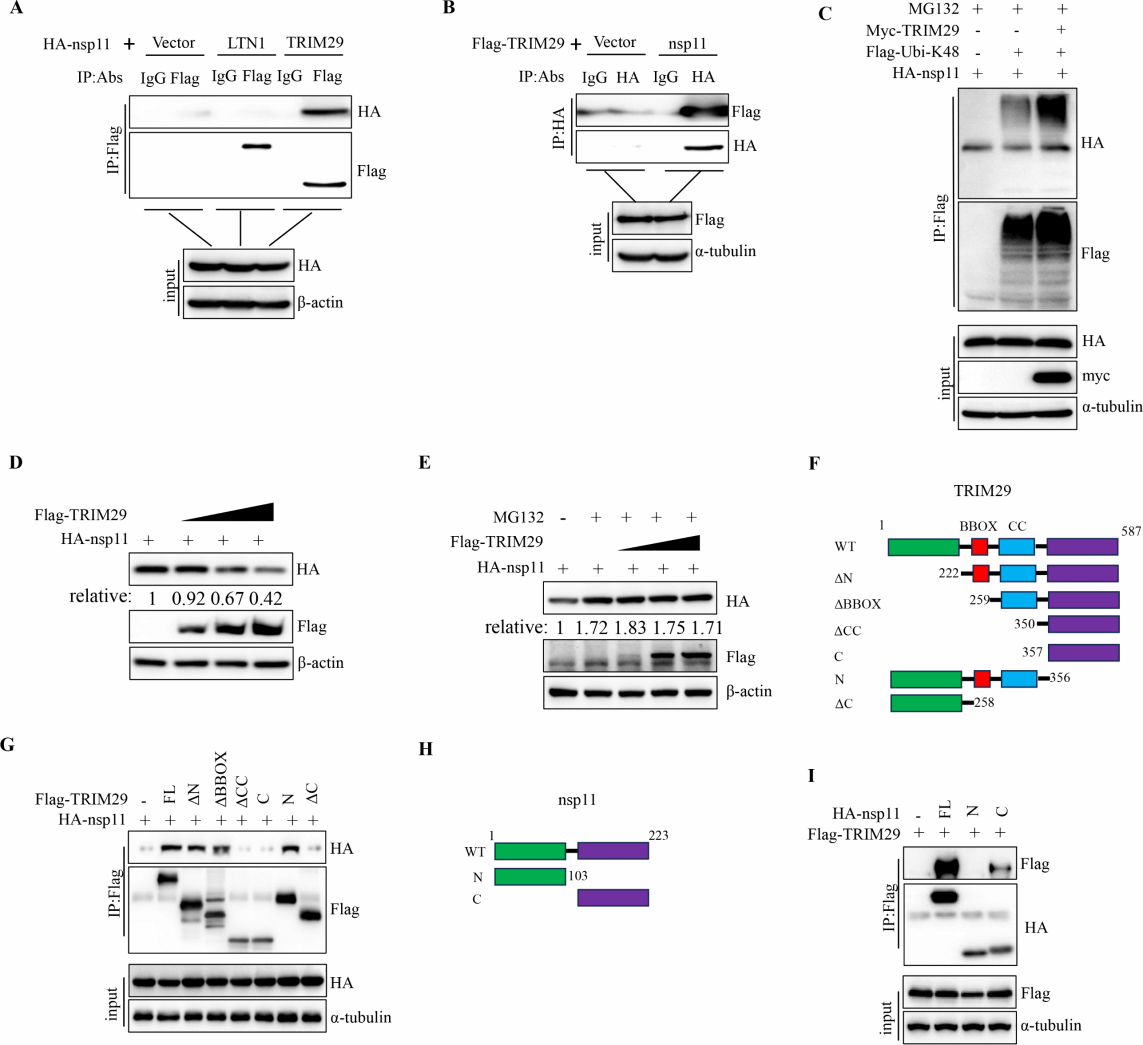
TRIM29 induces proteasomal degradation of PPRRSV nsp11 by promoting its K48-linked ubiquitination. (**A**) HEK293T cells were transfected with PRRSV nsp11 and vector, LTN1, or TRIM29 for 30 h, and then the cells were collected for immunoprecipitation, followed by western blot analysis of both total lysates and immunoprecipitated samples. (**B**) HEK293T cells were transfected with TRIM29 and vector or nsp11 for 30 h, and then the cells were collected for immunoprecipitation, followed by western blot analysis of both total lysates and immunoprecipitated samples. (**C**) HEK293T cells were transfected with vector or TRIM29 together with nsp11 and ubiquitin for 24 h, and then treated with MG132 for 10 h. The cells were collected for immunoprecipitation, followed by western blot analysis of both total lysates and immunoprecipitated samples. (**D and E**) HEK293T cells were transfected with nsp11 and different concentration TRIM29 for 24 h and then treated with 10 µM MG132 for 10 h. The cells were harvested for western blot. (**F**) Schematic representation of TRIM29 truncation mutants. (**G**) HEK293T cells were transfected with TRIM29 mutants and nsp11 for 30 h, and then the cells were collected for immunoprecipitation, followed by western blot analysis of both total lysates and immunoprecipitated samples. (**H**) Schematic representation of nsp11 truncation mutants. (**I**) HEK293T cells were transfected with nsp11 mutants and TRIM29 for 30 h, and then the cells were collected for immunoprecipitation, followed by western blot analysis of both total lysates and immunoprecipitated samples.

MS-based proteomic analysis revealed two E3 ubiquitin ligases, TRIM29 and LTN1, as potential interactors of PRRSV nsp11 (Fig. 6A). To validate these findings, co-immunoprecipitation (Co-IP) assays were performed, confirming a robust interaction between TRIM29 and nsp11 (Fig. 6A and B). Given the critical role of E3 ligases in regulating protein stability through ubiquitination, we next investigated whether TRIM29 modulates the ubiquitination status of nsp11. Strikingly, upon TRIM29 overexpression in the presence of the proteasome inhibitor MG132, we observed a pronounced increase in nsp11 ubiquitination (Fig. 6C), suggesting that TRIM29 mediates the polyubiquitination of nsp11, thereby targeting it for proteasomal degradation. Consistent with this hypothesis, TRIM29 overexpression led to a dose-dependent reduction in nsp11 protein levels (Fig. 6D and E), an effect that was abolished upon MG132 treatment, further supporting the notion that TRIM29 promotes nsp11 degradation via the ubiquitin-proteasome system. To illustrate the molecular determinants governing the TRIM29-nsp11 interaction, we systematically analyzed the binding capacity of nsp11 to various TRIM29 truncation mutants. Intriguingly, both recombinant full-length TRIM29 and its truncation mutants, except for ΔCC, C, or ΔC domain of TRIM29, interacted with nsp11, implicating the coiled-coil (CC) domain of TRIM29 as the critical region mediating this association (Fig. 6F and G). Conversely, reciprocal mapping using nsp11 truncation mutants demonstrated that both full-length nsp11 and its C-terminal domain were sufficient for TRIM29 binding (Fig. 6I). Furthermore, we assessed the potential interaction of TRIM29 with nsp11 proteins from other arteriviruses; however, no such interactions were detected (data not shown). Thus, even though arterivirus nsp11 is degraded by the host ubiquitin-proteasome system, the mechanisms are different. Collectively, these findings establish TRIM29 as a key regulator of nsp11 stability, driving its K48-linked polyubiquitination and subsequent proteasomal degradation.

### TRIM29 inhibits PRRSV replication

**Fig 7:**
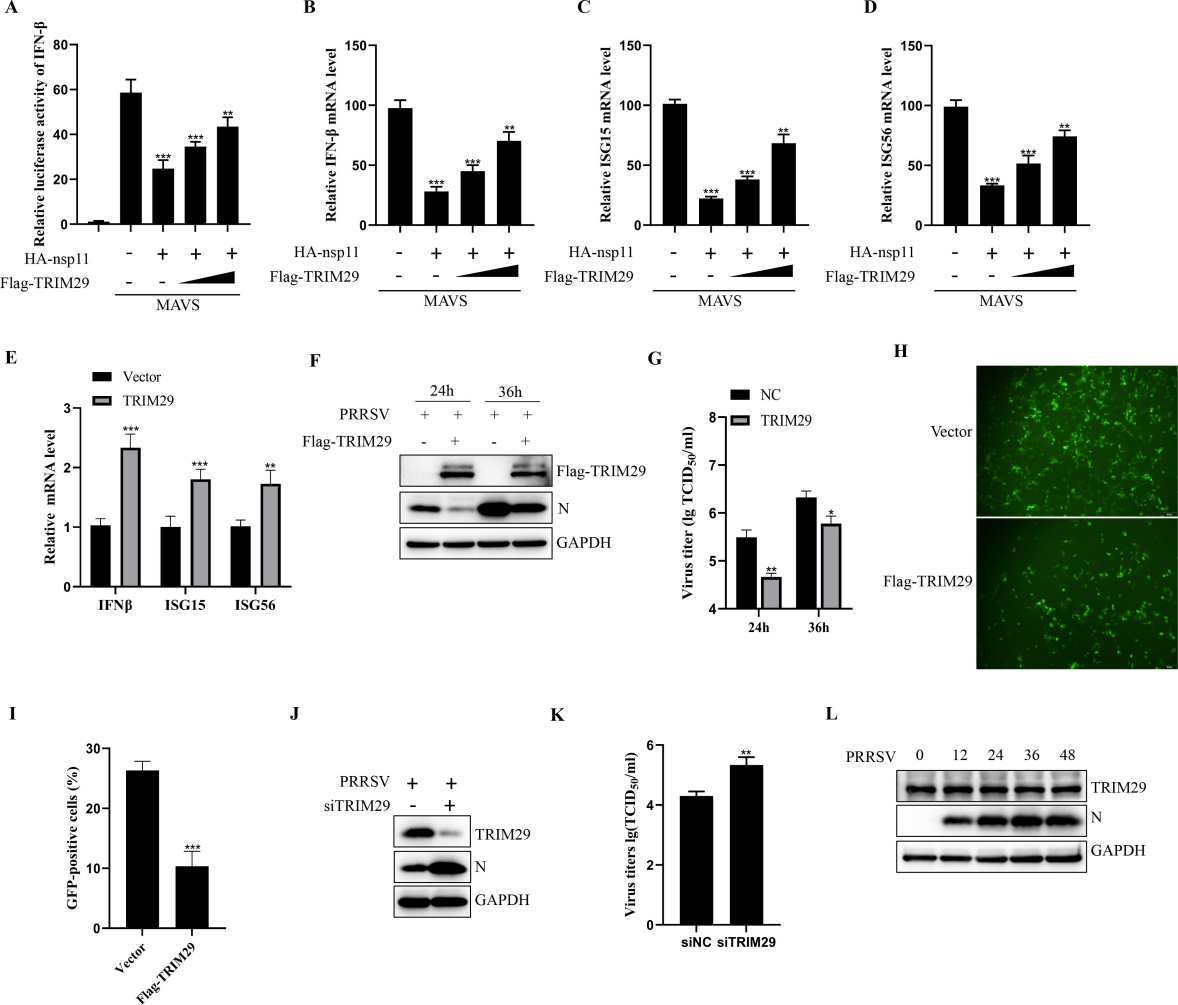
TRIM29 inhibits PRRSV replication. (**A**) HEK293T cells in 24-well plates were transfected with a firefly luciferase reporter plasmid and the indicated proteins. After 24 h, MAVS was transfected to activate the innate immune pathway. Luciferase activity was measured 24 h later to assess IFNβ promoter activity. (**B–D**) HEK293T cells carrying the indicated plasmids were transfected with MAVS for 24 h, followed by RT-qPCR analysis of IFNβ, ISG15, and ISG56 expression. (**E**) MARC145 cells, with or without TRIM29 overexpression, were infected with PRRSV. Antiviral gene expression was measured by RT-qPCR at 36 h post-infection. (**F and G**) MARC145 cells expressing vector or TRIM29 were infected with PRRSV (MOI = 0.1) for the indicated times and analyzed by western blot. Virus titers were determined by TCID_50_. (**H**) MARC145 cells expressing vector or TRIM29 were infected with PRRSV-GFP (MOI = 0.1) for 36 h, followed by fluorescence microscopy (scale bar = 50 µm). (**I**) Quantification of the percentage of GFP-positive cells from panel H. (**J and K**) PAM cells transfected with NC or siTRIM29 for 36 h were infected with PRRSV (MOI = 0.1) for 16 h. Cells were harvested for western blot, and virus titers were measured by TCID_50_. (**L**) MARC145 cells were infected with PRRSV for the indicated times and analyzed by western blot. *P < 0.05, **P < 0.01, and ***P < 0.001.

Previous studies have reported that nsp11 serves as a major IFN antagonist of PRRSV (19, 37). Given this finding, we sought to determine whether TRIM29 modulates nsp11-mediated suppression of IFN production. HEK293T cells were transfected with an IFN promoter-driven firefly luciferase reporter plasmid, along with nsp11 and TRIM29 (where applicable), while MAVS was employed as a strong inducer of IFN production. Consistent with prior reports, nsp11 overexpression significantly suppressed IFN-β production, but this inhibitory effect was markedly counteracted by TRIM29 co-expression (Fig. 7A). To further assess the impact of TRIM29 on IFN signaling, we measured the mRNA levels of IFN-β, ISG15, and ISG56 in HEK293T cells co-transfected with MAVS and either nsp11 alone or nsp11 plus TRIM29. Notably, TRIM29 promoted nsp11 degradation, leading to higher mRNA levels of IFN-β and interferon-stimulated genes (ISGs) compared to cells expressing nsp11 alone (Fig. 7B through D). Moreover, PRRSV infection in TRIM29-overexpressing cells significantly upregulated antiviral cytokines, including IFN-β, ISG15, and ISG56 (Fig. 7E). These findings suggest that TRIM29 antagonizes nsp11-mediated IFN suppression by enhancing its degradation.

To explore TRIM29’s role in PRRSV replication, we examined its effects on viral protein expression and infectivity. Overexpression of TRIM29 significantly reduced viral N protein levels and virus titers (Fig. 7F and G). To confirm these observations, vector-expressing or TRIM29-expressing cells were infected with PRRSV-GFP for 36 h. Compared to control cells, which exhibited strong fluorescence, TRIM29-expressing cells displayed substantially weaker GFP signals, further supporting its antiviral role (Fig. 7H and I). Conversely, small interfering RNA (siRNA)-mediated knockdown of TRIM29 in PAM cells enhanced N protein expression and viral titers at 16 h post-infection (Fig. 7J and K), reinforcing the notion that TRIM29 restricts PRRSV replication. To determine whether PRRSV infection influences TRIM29 expression, we infected MARC-145 cells with PRRSV (MOI = 0.1) and analyzed TRIM29 levels by Western blotting at various time points. However, PRRSV infection did not significantly alter TRIM29 expression (Fig. 7L).

## DISCUSSION

Ubiquitination, a pivotal post-translational modification, regulates critical cellular processes including protein homeostasis, innate immunity, and antiviral defense (38). To evade immune surveillance, viruses encode specialized proteins that exploit the host ubiquitination machinery. These viral effectors selectively target host antiviral defenses, such as restriction factors and key signaling molecules (e.g., MAVS and NF-κB), ultimately suppressing innate immune activation (39, 40). Mechanistically, certain viral proteins achieve immune evasion by either removing ubiquitin modifications from RIG-I and STING or blocking their interactions with cognate E3 ligases, thereby impairing their activation (41–43). Conversely, the host counteracts viral infection by mobilizing E3 ubiquitin ligases to mediate proteasomal degradation of viral proteins through the UPS, effectively attenuating viral virulence (21, 44, 45). The present study uncovers a previously unrecognized antiviral mechanism wherein the host E3 ubiquitin ligase TRIM29 selectively targets the conserved endoribonuclease nsp11 of PRRSV for K48-linked polyubiquitination and subsequent proteasomal degradation. Our findings provide key mechanistic insights into how host cells restrict arteriviral replication by exploiting the vulnerability of a catalytically essential residue within a critical viral enzyme. This work highlights a unique interface between host ubiquitination machinery and viral enzymatic function, offering conceptual and therapeutic implications for the broader control of arteriviral infections.

A central discovery of this study is that PRRSV nsp11 is ubiquitinated at lysine 173 (K173), a residue indispensable for its endoribonuclease activity (Fig. 2C and 4B). Notably, nsp11 K173 site is not only sufficient to trigger proteasomal degradation (Fig. 5A and F) but also structurally coupled to enzymatic inactivation (37, 46). These findings align with emerging evidence that host cells recognize and destabilize conserved viral enzymatic motifs as a defense strategy. The selective pressure imposed by host ubiquitination at K173 may explain the high conservation of this residue across PRRSV strains. Furthermore, our evolutionary analyses show that this regulatory mechanism is conserved across multiple arterivirus species, including SHFV and EAV, despite sequence divergence (Fig. 4C and D). However, LDV is a notable exception, as it appears to use an alternative degradation strategy (Fig. 4E). This conserved targeting of the catalytic site suggests an evolutionary “Achilles’ heel” that the host exploits to restrict a broad spectrum of arteriviruses. Mechanistically, we identify TRIM29 as the host factor responsible for directing the K48-linked polyubiquitination of PRRSV nsp11. Our data show that TRIM29 binds nsp11 via its CC domain, a region involved in substrate recognition for immune regulators like STING and NEMO. This finding underscores TRIM29’s broader role in innate immune regulation. The specificity of TRIM29 for PRRSV nsp11, and not for nsp11 homologs from other arteriviruses, suggests that TRIM29-substrate recognition is likely determined by virus-specific structural or contextual motifs within the C-terminal region of nsp11.

Importantly, we show that TRIM29-mediated degradation of nsp11 reactivates the antiviral IFN pathway by relieving nsp11-mediated suppression of IFN-β and ISGs (Fig. 7A through E). Given that nsp11 has been previously established as a potent antagonist of MAVS-dependent signaling (36), its removal by TRIM29 shifts the balance in favor of host defense. Indeed, overexpression of TRIM29 leads to significant reductions in viral protein synthesis and virus titers, and TRIM29 knockdown enhances PRRSV replication (Fig. 7F through J).

From a broader perspective, our results establish a precedent for host-targeted degradation of viral enzymes via modification of their catalytic cores, which is an elegant strategy that circumvents the need for host recognition of structurally variable epitopes. The fact that TRIM29 modifies a functionally irreplaceable lysine within nsp11’s active site provides a compelling example of post-translational regulation functioning as a molecular switch for viral attenuation. This discovery not only expands our understanding of host-pathogen conflict at the interface of ubiquitin biology and RNA virus replication but also suggests novel antiviral strategies. Small molecules or gene therapy approaches that mimic or enhance TRIM29 activity could represent promising therapeutic avenues against PRRSV.

Finally, our findings raise intriguing questions about viral countermeasures. It remains to be determined whether PRRSV encodes antagonists that neutralize TRIM29 or otherwise prevent nsp11 degradation. While our data indicate that PRRSV infection does not modulate TRIM29 expression at the protein level, post-translational modifications or spatial sequestration could still underlie potential evasion strategies. Future investigations are warranted to explore the dynamic interplay between TRIM29, viral proteins, and other cellular quality control pathways.

In summary, this work defines a previously unappreciated host-driven restriction mechanism targeting the catalytic core of a key viral enzyme, mediated by TRIM29-dependent ubiquitination. It sheds light on a conserved vulnerability in arterivirus replication machinery and opens new avenues for host-directed antiviral interventions.

## MATERIALS AND METHODS

### Cells and virus

HEK-293T cells (ATCC CRL-11268) and MARC-145 cells (African green monkey kidney cells; ATCC CRL-12231) were maintained in Dulbecco’s modified Eagle’s medium (HyClone, SH30022.01) supplemented with 10% fetal bovine serum (Lonsa Science, S711-001S) and 1% penicillin-streptomycin (Solarbio, P1400). PAMs and the PRRSV strain were preserved in our laboratory. The PRRSV-EGFP recombinant virus, which stably expresses enhanced green fluorescent protein (EGFP), was generously provided by Dr. Nanhua Chen (Yangzhou University).

### Plasmids, antibodies, and reagents

Plasmids encoding PRRSV nsp11 and its mutant variants were constructed and stored in our laboratory. TRIM29 was amplified from MARC-145 cell-derived cDNA using gene-specific primers, and site-directed mutagenesis was performed to generate TRIM29 mutants. All constructs, including wild-type and mutant TRIM29, were cloned into the pCDNA3.1-3Flag vector (Miaolingbio, P0157) using T4 DNA ligase (Promega, M1801). Additionally, nsp11 genes from SHFV, LDV, and EAV, along with their respective mutants, were synthesized by Genewiz and inserted into the same vector backbone.

Primary antibodies, including mouse anti-HA (66006-2-Ig), rabbit anti-HA (51064-2-AP), mouse anti-Flag (66008-3-Ig), and mouse anti-α-tubulin (6031-1-Ig), were purchased from Proteintech. Secondary antibodies conjugated to Alexa Fluor 555 (goat anti-mouse, Invitrogen, A32727) and Alexa Fluor 488 (goat anti-rabbit, A32731) were purchased from Invitrogen. Rabbit anti-TRIM29 polyclonal antibody (Abclonal, A5476) was used for TRIM29 detection. The anti-PRRSV N monoclonal antibody was developed and preserved in our laboratory.

Chemical reagents included chloroquine (autophagy inhibitor; Beyotime, C1202) and MG132 (proteasome inhibitor; Selleck, S2619).

### Plasmid transfection

HEK293T and MARC-145 cells were transiently transfected with plasmid DNA using JetPRIME transfection reagent (Polyplus, PT-114-15), according to the manufacturer’s instructions.

### RNA interference

siRNAs targeting specific genes were designed and synthesized by GenePharma. PAM cells were transfected with siRNAs at a final concentration of 10 nM using Lipofectamine 2000 (Invitrogen, 11668019), following the manufacturer’s protocol. Cells were infected with PRRSV 36 h post-transfection.

### RT-qPCR analysis

Total RNA was extracted using TRIzol reagent (Invitrogen) and reverse-transcribed into cDNA using the HiScript III 1st Strand cDNA Synthesis Kit (Vazyme, R312). Quantitative PCR was performed using ChamQ Universal SYBR qPCR Master Mix (Vazyme, Q711). Relative gene expression levels were calculated using the 2^−ΔΔCt^ method.

### Dual-luciferase reporter assay

HEK293T cells were seeded in 24-well plates 24 h prior to transfection. Cells were co-transfected with an IFN-β luciferase reporter plasmid and pTK-Renilla, along with the indicated expression plasmids. An empty vector was included as a negative control to equalize the total plasmid DNA amount. Luciferase activities were measured using a Dual-Luciferase Reporter Assay System (Beyotime, RG027) according to the manufacturer’s protocol. Each experiment was performed in triplicate. Results are presented as mean ± standard deviation.

### Western blotting, Co-IP, and LC-MS/MS

Cells were lysed in lysis buffer and incubated on ice for 30 min with intermittent vortexing. Lysates were clarified by centrifugation at 12,000 rpm for 10 min. Proteins were separated by SDS-PAGE and transferred onto PVDF membranes (Roche, 46978100). Membranes were blocked in 5% skim milk and incubated with primary antibodies for 1 h at room temperature, followed by secondary antibody incubation.

For Co-IP analysis, cell lysates were incubated overnight at 4°C with specific antibodies, then mixed with Protein A+G agarose beads (Beyotime, P2012) and further incubated at 4°C for 3 h. The resulting complexes were collected by centrifugation at 1,000 × *g* for 3 min, washed five times with cold lysis buffer, and analyzed by Western blotting.

For LC-MS/MS analysis, immunoprecipitated complexes were resolved via SDS-PAGE, followed by silver staining to visualize differentially expressed protein bands between experimental and control groups. Selected bands were excised and subjected to LC-MS/MS by APTBIO (Shanghai, China) to identify nsp11-interacting proteins.

### Statistical analysis

All data were analyzed using GraphPad Prism 9. Each experiment was independently repeated at least three times. Statistical comparisons between two groups were performed using a two-tailed Student’s *t*-test. A *P* value <0.05 was considered statistically significant (*P < 0.05, **P < 0.01, and ***P < 0.001).

## Data Availability

The data that support the findings of this study are available within the article.

## References

[1] Han M, Yoo D. 2014. Engineering the PRRS virus genome: updates and perspectives. Vet Microbiol 174:279–295. doi:10.1016/j.vetmic.2014.10.007 25458419 PMC7172560

[2] Music N, Gagnon CA. 2010. The role of porcine reproductive and respiratory syndrome (PRRS) virus structural and non-structural proteins in virus pathogenesis. Anim Health Res Rev 11:135–163. doi:10.1017/S1466252310000034 20388230

[3] Zhong Y, Tan YW, Liu DX. 2012. Recent progress in studies of arterivirus- and coronavirus-host interactions. Viruses 4:980–1010. doi:10.3390/v4060980 22816036 PMC3397358

[4] Snijder EJ, Kikkert M, Fang Y. 2013. Arterivirus molecular biology and pathogenesis. J Gen Virol 94:2141–2163. doi:10.1099/vir.0.056341-0 23939974

[5] Shaw TM, Huey D, Mousa-Makky M, Compaleo J, Nennig K, Shah AP, Jiang F, Qiu X, Klipsic D, Rowland RRR, Slukvin II, Sullender ME, Baldridge MT, Li H, Warren CJ, Bailey AL. 2024. The neonatal Fc receptor (FcRn) is a pan-arterivirus receptor. Nat Commun 15:6726. doi:10.1038/s41467-024-51142-x 39112502 PMC11306234

[6] Han J, Zhou L, Ge X, Guo X, Yang H. 2017. Pathogenesis and control of the Chinese highly pathogenic porcine reproductive and respiratory syndrome virus. Vet Microbiol 209:30–47. doi:10.1016/j.vetmic.2017.02.020 28292547

[7] Chai W, Liu Z, Sun Z, Su L, Zhang C, Huang L. 2020. Efficacy of two porcine reproductive and respiratory syndrome (PRRS) modified-live virus (MLV) vaccines against heterologous NADC30-like PRRS virus challenge. Vet Microbiol 248:108805. doi:10.1016/j.vetmic.2020.108805 32828938

[8] Bai X, Wang Y, Xu X, Sun Z, Xiao Y, Ji G, Li Y, Tan F, Li X, Tian K. 2016. Commercial vaccines provide limited protection to NADC30-like PRRSV infection. Vaccine (Auckl) 34:5540–5545. doi:10.1016/j.vaccine.2016.09.048 27712853

[9] Chen X, Zhou X, Guo T, Qiao S, Guo Z, Li R, Jin Q, Hu X, Xing G, Deng R, Wan B, Zhang G. 2021. Efficacy of a live attenuated highly pathogenic PRRSV vaccine against a NADC30-like strain challenge: implications for ADE of PRRSV. BMC Vet Res 17:260. doi:10.1186/s12917-021-02957-z 34332554 PMC8325048

[10] Gao ZQ, Guo X, Yang HC. 2004. Genomic characterization of two Chinese isolates of porcine respiratory and reproductive syndrome virus. Arch Virol 149:1341–1351. doi:10.1007/s00705-004-0292-0 15221535

[11] Dokland T. 2010. The structural biology of PRRSV. Virus Res 154:86–97. doi:10.1016/j.virusres.2010.07.029 20692304 PMC7114433

[12] Fang Y, Snijder EJ. 2010. The PRRSV replicase: exploring the multifunctionality of an intriguing set of nonstructural proteins. Virus Res 154:61–76. doi:10.1016/j.virusres.2010.07.030 20696193 PMC7114499

[13] Ivanov KA, Hertzig T, Rozanov M, Bayer S, Thiel V, Gorbalenya AE, Ziebuhr J. 2004. Major genetic marker of nidoviruses encodes a replicative endoribonuclease. Proc Natl Acad Sci USA 101:12694–12699. doi:10.1073/pnas.0403127101 15304651 PMC514660

[14] Laneve P, Altieri F, Fiori ME, Scaloni A, Bozzoni I, Caffarelli E. 2003. Purification, cloning, and characterization of XendoU, a novel endoribonuclease involved in processing of intron-encoded small nucleolar RNAs in Xenopus laevis. J Biol Chem 278:13026–13032. doi:10.1074/jbc.M211937200 12571235

[15] Ricagno S, Egloff MP, Ulferts R, Coutard B, Nurizzo D, Campanacci V, Cambillau C, Ziebuhr J, Canard B. 2006. Crystal structure and mechanistic determinants of SARS coronavirus nonstructural protein 15 define an endoribonuclease family. Proc Natl Acad Sci USA 103:11892–11897. doi:10.1073/pnas.0601708103 16882730 PMC2131687

[16] Shi Y, Li Y, Lei Y, Ye G, Shen Z, Sun L, Luo R, Wang D, Fu ZF, Xiao S, Peng G. 2016. A dimerization-dependent mechanism drives the endoribonuclease function of porcine reproductive and respiratory syndrome virus nsp11. J Virol 90:4579–4592. doi:10.1128/JVI.03065-15 26912626 PMC4836315

[17] Wang R, Zhang YJ. 2014. Antagonizing interferon-mediated immune response by porcine reproductive and respiratory syndrome virus. Biomed Res Int 2014:315470. doi:10.1155/2014/315470 25101271 PMC4101967

[18] Yang L, Zhang YJ. 2017. Antagonizing cytokine-mediated JAK-STAT signaling by porcine reproductive and respiratory syndrome virus. Vet Microbiol 209:57–65. doi:10.1016/j.vetmic.2016.12.036 28069291 PMC7117332

[19] Wang D, Chen J, Yu C, Zhu X, Xu S, Fang L, Xiao S. 2019. Porcine reproductive and respiratory syndrome virus nsp11 antagonizes Type I interferon signaling by targeting IRF9. J Virol 93. doi:10.1128/JVI.00623-19 PMC6639278 31092569

[20] Komander D. 2009. The emerging complexity of protein ubiquitination. Biochem Soc Trans 37:937–953. doi:10.1042/BST0370937 19754430

[21] Li Z, Hao P, Zhao Z, Gao W, Huan C, Li L, Chen X, Wang H, Jin N, Luo ZQ, Li C, Zhang W. 2023. The E3 ligase RNF5 restricts SARS-CoV-2 replication by targeting its envelope protein for degradation. Sig Transduct Target Ther 8:53. doi:10.1038/s41392-023-01335-5 PMC9897159 36737599

[22] Castañeda CA, Chaturvedi A, Camara CM, Curtis JE, Krueger S, Fushman D. 2016. Linkage-specific conformational ensembles of non-canonical polyubiquitin chains. Phys Chem Chem Phys 18:5771–5788. doi:10.1039/c5cp04601g 26422168 PMC4758893

[23] Buetow L, Huang DT. 2016. Structural insights into the catalysis and regulation of E3 ubiquitin ligases. Nat Rev Mol Cell Biol 17:626–642. doi:10.1038/nrm.2016.91 27485899 PMC6211636

[24] Velez-Brochero M, Behera P, Afreen KS, Odle A, Rajsbaum R. 2024. Ubiquitination in viral entry and replication: mechanisms and implications. Adv Virus Res 119:1–38. doi:10.1016/bs.aivir.2024.05.001 38897707

[25] van Gent M, Sparrer KMJ, Gack MU. 2018. TRIM proteins and their roles in antiviral host defenses. Annu Rev Virol 5:385–405. doi:10.1146/annurev-virology-092917-043323 29949725 PMC6186430

[26] Hage A, Rajsbaum R. 2019. To TRIM or not to TRIM: the balance of host-virus interactions mediated by the ubiquitin system. J Gen Virol 100:1641–1662. doi:10.1099/jgv.0.001341 31661051 PMC7011758

[27] Ganser-Pornillos BK, Pornillos O. 2019. Restriction of HIV-1 and other retroviruses by TRIM5. Nat Rev Microbiol 17:546–556. doi:10.1038/s41579-019-0225-2 31312031 PMC6858284

[28] Fan W, Mar KB, Sari L, Gaszek IK, Cheng Q, Evers BM, Shelton JM, Wight-Carter M, Siegwart DJ, Lin MM, Schoggins JW. 2021. TRIM7 inhibits enterovirus replication and promotes emergence of a viral variant with increased pathogenicity. Cell 184:3410–3425. doi:10.1016/j.cell.2021.04.047 34062120 PMC8276836

[29] Lin L, Wang X, Chen Z, Deng T, Yan Y, Dong W, Huang Y, Zhou J. 2023. TRIM21 restricts influenza A virus replication by ubiquitination-dependent degradation of M1. PLoS Pathog 19:e1011472. doi:10.1371/journal.ppat.1011472 37343022 PMC10325077

[30] Xing J, Zhang A, Zhang H, Wang J, Li XC, Zeng MS, Zhang Z. 2017. TRIM29 promotes DNA virus infections by inhibiting innate immune response. Nat Commun 8:945. doi:10.1038/s41467-017-00101-w 29038422 PMC5643338

[31] Wu Q, Nandi D, Sharma D. 2024. TRIM-endous functional network of tripartite motif 29 (TRIM29) in cancer progression and beyond. Cancer Metastasis Rev 44:16. doi:10.1007/s10555-024-10226-2 39644332 PMC11625080

[32] Li Q, Lin L, Tong Y, Liu Y, Mou J, Wang X, Wang X, Gong Y, Zhao Y, Liu Y, Zhong B, Dai L, Wei YQ, Zhang H, Hu H. 2018. TRIM29 negatively controls antiviral immune response through targeting STING for degradation. Cell Discov 4:13. doi:10.1038/s41421-018-0010-9 29581886 PMC5859251

[33] Xing J, Weng L, Yuan B, Wang Z, Jia L, Jin R, Lu H, Li XC, Liu YJ, Zhang Z. 2016. Identification of a role for TRIM29 in the control of innate immunity in the respiratory tract. Nat Immunol 17:1373–1380. doi:10.1038/ni.3580 27695001 PMC5558830

[34] Nisole S, Stoye JP, Saïb A. 2005. TRIM family proteins: retroviral restriction and antiviral defence. Nat Rev Microbiol 3:799–808. doi:10.1038/nrmicro1248 16175175

[35] Randow F, Lehner PJ. 2009. Viral avoidance and exploitation of the ubiquitin system. Nat Cell Biol 11:527–534. doi:10.1038/ncb0509-527 19404332

[36] Sun Y, Ke H, Han M, Chen N, Fang W, Yoo D. 2016. Nonstructural protein 11 of porcine reproductive and respiratory syndrome virus suppresses both MAVS and RIG-I expression as one of the mechanisms to antagonize type i interferon production. PLoS One 11:e0168314. doi:10.1371/journal.pone.0168314 27997564 PMC5172586

[37] Shi X, Wang L, Li X, Zhang G, Guo J, Zhao D, Chai S, Deng R. 2011. Endoribonuclease activities of porcine reproductive and respiratory syndrome virus nsp11 was essential for nsp11 to inhibit IFN-β induction. Mol Immunol 48:1568–1572. doi:10.1016/j.molimm.2011.03.004 21481939 PMC7112683

[38] Valerdi KM, Hage A, van Tol S, Rajsbaum R, Giraldo MI. 2021. The role of the host ubiquitin system in promoting replication of emergent viruses. Viruses 13:369. doi:10.3390/v13030369 33652634 PMC7996891

[39] Gu H, Jan Fada B. 2020. Specificity in ubiquitination triggered by virus infection. Int J Mol Sci 21:4088. doi:10.3390/ijms21114088 32521668 PMC7313089

[40] Liu B, Zhang M, Chu H, Zhang H, Wu H, Song G, Wang P, Zhao K, Hou J, Wang X, Zhang L, Gao C. 2017. The ubiquitin E3 ligase TRIM31 promotes aggregation and activation of the signaling adaptor MAVS through Lys63-linked polyubiquitination. Nat Immunol 18:214–224. doi:10.1038/ni.3641 27992402

[41] Fan Y, Mao R, Yu Y, Liu S, Shi Z, Cheng J, Zhang H, An L, Zhao Y, Xu X, Chen Z, Kogiso M, Zhang D, Zhang H, Zhang P, Jung JU, Li X, Xu G, Yang J. 2014. USP21 negatively regulates antiviral response by acting as a RIG-I deubiquitinase. J Exp Med 211:313–328. doi:10.1084/jem.20122844 24493797 PMC3920558

[42] Zhou Y, Zheng R, Liu D, Liu S, Disoma C, Li S, Liao Y, Chen Z, Du A, Dong Z, et al.. 2022. UBR5 acts as an antiviral host factor against MERS-CoV via promoting ubiquitination and degradation of ORF4b. J Virol 96:e0074122. doi:10.1128/jvi.00741-22 35980206 PMC9472757

[43] Bodda C, Reinert LS, Fruhwürth S, Richardo T, Sun C, Zhang B-C, Kalamvoki M, Pohlmann A, Mogensen TH, Bergström P, Agholme L, O’Hare P, Sodeik B, Gyrd-Hansen M, Zetterberg H, Paludan SR. 2020. HSV1 VP1-2 deubiquitinates STING to block type I interferon expression and promote brain infection. J Exp Med 217:e20191422. doi:10.1084/jem.20191422 32383759 PMC7336311

[44] Yu X, Chen S, Hou P, Wang M, Chen Y, Guo D. 2015. VHL negatively regulates SARS coronavirus replication by modulating nsp16 ubiquitination and stability. Biochem Biophys Res Commun 459:270–276. doi:10.1016/j.bbrc.2015.02.097 25732088 PMC7092858

[45] Wang Y, Dong Y, Luan T, Chen Y, Lin L, Li S, Feng D, Wei J, Fei Y, Wang G, Pan J, Wang Y, Zhong Z, Zhao W. 2024. TRIM56 restricts Coxsackievirus B infection by mediating the ubiquitination of viral RNA-dependent RNA polymerase 3D. PLoS Pathog 20:e1012594. doi:10.1371/journal.ppat.1012594 39348396 PMC11476688

[46] Dong H, Zhou L, Ge X, Guo X, Han J, Yang H. 2018. Porcine reproductive and respiratory syndrome virus nsp1β and nsp11 antagonize the antiviral activity of cholesterol-25-hydroxylase via lysosomal degradation. Vet Microbiol 223:134–143. doi:10.1016/j.vetmic.2018.08.012 30173739

